# The Origins of Engineered Biomaterials: NSF-Funded, University of Washington Engineered Biomaterials (UWEB) †

**DOI:** 10.3390/bioengineering11111117

**Published:** 2024-11-06

**Authors:** Buddy D. Ratner

**Affiliations:** 1 Department of Bioengineering, University of Washington, Seattle, WA 98195, USA; 2 Department of Chemical Engineering, University of Washington, Seattle, WA 98195, USA

**Keywords:** biocompatibility, healing, engineered biomaterials, foreign body reaction, engineering research center

## Abstract

The University of Washington Engineered Biomaterials (UWEB) Engineering Research Center (ERC) was funded from 1996 to 2007 by the U.S. National Science Foundation. The mission of UWEB was to advance biomaterials by integrating modern biology with materials science. UWEB specifically focused on the healing and integration of medical implants. UWEB teamed biologists, physicians, engineers, and industry and demonstrated three paths that might advance biomaterials so they could seamlessly integrate and heal in the body. The three primary lines of investigation were precision porous scaffolds, super-non-fouling surfaces, and the control of matricellular proteins. The UWEB program set the groundwork for the modern field of immunoengineering. Also, UWEB invested significantly in training scientists/engineers who could freely integrate advances in biological sciences, state-of-the-art materials science, and medical technology. This historical summary of the UWEB program demonstrates that federal investment in interfacing forefront fields can yield dividends with benefits for society and the economy.

## 1. Setting the Stage

The University of Washington Engineered Biomaterials (UWEB) Engineering Research Center (ERC) was funded by the U.S. National Science Foundation (NSF) from 1996 to 2007. I served as the Director of the UWEB Center through its NSF-funded lifetime. The thinking that led to UWEB is an important part of this story, and the outcomes from UWEB have had a significant impact on contemporary biomaterials research. This historical overview and personal recollection will tell the story of UWEB’s origins, UWEB contributions, and UWEB’s impact on the field of biomaterials.

### 1.1. The Making of UWEB

This article is primarily about the UWEB ERC, but the path that led to the UWEB was convoluted and interesting. The story of the roots of UWEB will be told, and then the UWEB Center and its output will be described.

I started biomaterials research at Brooklyn Polytechnic Institute in 1967 (incidentally, the first use of the word “biomaterial” that I could find in the scientific literature was about 1965). In 1972, I was recruited by Professor Allan S. Hoffman to come to the University of Washington (UW) to explore materials that would be used in contact with blood in an Atomic Energy Commission (AEC)-sponsored nuclear-powered artificial heart. From the beginning, our University of Washington lab had a strong focus on interfacial proteins, cell interactions with surfaces, and, ultimately, the reaction of the body to implanted materials. In our lab at that time, and starting his climb up the academic ladder, was Dr. Thomas Horbett. In one of the earliest proposals that Tom and I submitted to the National Institutes of Health (NIH) in roughly 1976, we asked a question: Polyethylene and gold obviously have different surface properties and adsorb different proteins. So, why do polyethylene and gold both produce almost indistinguishable foreign body reactions (FBR)? The FBR to these two materials is characterized by a thin, relatively avascular, acellular collagenous capsule. The grant was funded (“Cell Interactions with Materials”) and was refunded many times over the years. This funded research gave us a platform to explore interfacial proteins and cell–surface interactions. We learned and published much on these subjects over the years but did not resolve the gold–polyethylene conundrum.

One lesson from our studies under the NIH Cell Interaction grant was that we must become much better at characterizing the surfaces we used in these explorations. For example, it is easy to think of polyethylene as simply a carbon–hydrogen polymer. But, as we later found out, every sample of polyethylene we examined had varying levels of oxygen in the outermost surface zone, and oxygen was distributed through a range of carbon–hydrogen–oxygen functional groups (ester, ether, alcohol, acid, peroxide, etc.). The pressure to bring meaningful surface characterization to biomaterials led me to explore what techniques were available to assess surface properties. Contact angle measurements were widely used at the time to assess “wettability”. A change in the contact angle will correspond to a change in surface chemistry, but it was challenging to translate that observation into the identification of specific functional groups. We had in our lab a World War II-era infrared (IR) spectrometer. The attenuated total reflectance (ATR-IR) method could give more specific chemical information, but its penetration depth into the surface was microns, and we felt all the action was happening in the outermost few angstroms, the near surface. Also, the instrument was painfully slow—IR did not become a practical means for surface characterization until the advent of the Fourier Transform IR instruments in the early 1980s.

**Figure 1:**
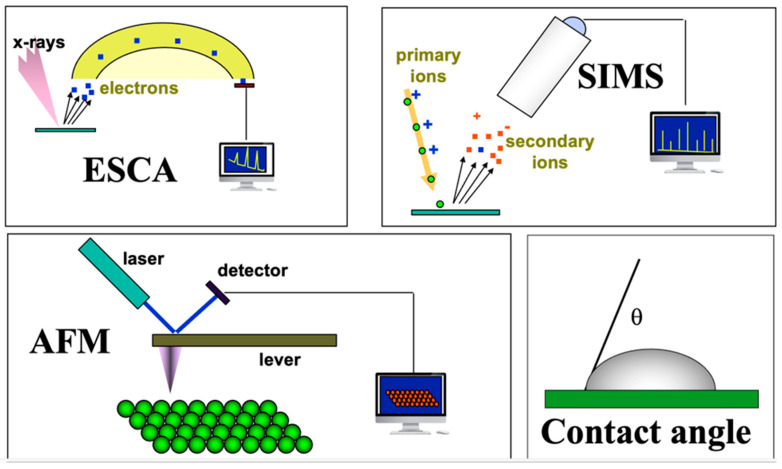
Surface analytical tools applied at the National ESCA and Surface Analysis Center for Biomedical Problems (NESAC/BIO), University of Washington, Seattle, WA, USA. Electron spectroscopy for chemical analysis (ESCA, also called XPS), secondary ion mass spectrometry (SIMS). Atomic force microscopy (AFM) and contact angle methods.

In the mid-1970s, a relatively new technique, electron spectroscopy for chemical analysis (ESCA), had transitioned from the province of fundamental studies on the physics of solid-state materials to a useful analytical method. The ESCA method was then used primarily for studying electron transitions in atoms and also for characterizing petrochemical catalysts. ESCA was highly surface sensitive and rich in chemical information… and complicated… and expensive! In collaboration with physicists and engineers at Hewlett Packard Corporation in Palo Alto, California, we began exploring biomaterial surfaces. This quickly led to several significant discoveries about the surface structure and composition of biomaterials. Thanks to a gift from the Shell Foundations to our Chemical Engineering department, we were able to purchase our own ESCA instrument, and I used this as the core instrument in an NIH P41 grant application requesting funds to establish a national resource center devoted to the surface characterization of biomaterials. This was funded in 1984 and branded as the National ESCA and Surface Analysis Center for Biomedical Problems (NESAC/BIO). Over the years, other characterization tools, such as secondary ion mass spectrometry (SIMS), were added to NESAC/BIO (Figure 1). We had the ability to characterize biomaterial surfaces with considerable precision. The surface information from these methods provided insights into blood compatibility and helped us understand certain adverse reactions to implanted medical devices. However, the surface information still did not reveal why gold and polyethylene, two such different materials, healed so similarly in the body. In fact, by that time, we had examined the soft tissue healing of numerous materials: hydrophilic, hydrophobic, charged, neutral, hard, soft, etc. As long as the materials did not leach cytotoxic substances, they all healed almost indistinguishably, surrounded by a thin, relatively avascular, collagenous capsule.

In 1992, I attended a conference organized by the Polymer Division of the American Chemical Society (POLY), Polymers in Medicine and Biology, held in Palm Springs, California, and chaired by Professor James Anderson. I heard a lecture delivered by Dr. James Brauker of Baxter Corporation that opened my eyes to the possibility that the FBR might not be the inevitable outcome for implants in the body. Dr. Brauker described animal implantation studies of a large number of commercially sourced, porous polymeric materials. Some of the materials in this set healed with low fibrosis and new vascularity, while others showed the classic FBR with a dense, fibrotic capsule. It was not clear why some materials induced reconstructive healing and others did not, but these experiments did demonstrate that implant reactions to materials were not confined to the classic FBR.

This insight, that more desirable healing responses might be orchestrated, led me to write a perspective article suggesting that we could move biomaterials into a less fibrotic and more pro-regenerative space [1]. The article received much attention (it now has 626 citations). One person who noticed that article was my department chair, Professor Lee Huntsman. I recall being at a conference that was also attended by Lee. As I descended the podium after a talk expounding a vision for next-generation biomaterials, Lee approached me with a paper in hand. It was a call for proposals for the establishment of NSF ERCs. Lee’s words to me were clear—you’ve got to go from “talking the talk to walking the walk”. It was time to take the ideas of biomaterials that direct healing and biological responses from a vision to a reality, and the NSF ERCs presented a platform to make that happen. The University of Washington was in a prime position to execute this vision. The Department of Bioengineering resided administratively between the College of Engineering and the School of Medicine. We were well poised to tap into both academic camps for bringing multidisciplinary thinking to this project.

Since the 1960s, the University of Washington has played a leading role on the world stage in understanding injury, healing, and inflammation, taking these subjects from phenomenology to a mechanistic understanding. First, under Dr. Earl Benditt and then under Dr. Russell Ross, molecular biology concepts were aimed toward the study of pathological conditions, tissue injury, and healing [2,3,4]. A community of outstanding molecular biologists focusing on these subjects coalesced around these pioneers. This was the rich pool of medicine/biology talent that the nascent ERC tapped into. The College of Engineering at the University of Washington offered yet another talent pool with relevant players in Bioengineering, Chemical Engineering, and Materials Science and Engineering.

### 1.2. The University of Washington Engineered Biomaterials (UWEB) ERC

**Figure 2:**
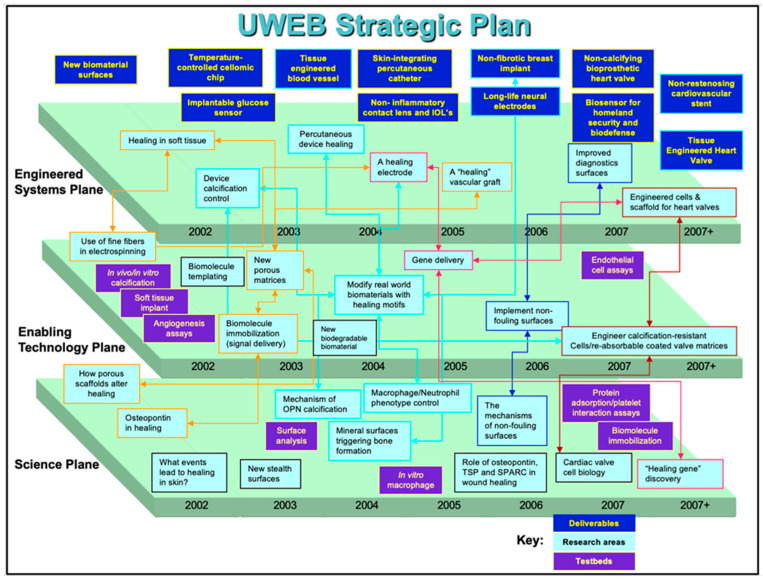
The UWEB Strategic Plan 2002–2007.

In 1995, our team submitted a pre-proposal to the NSF, competing with 117 other applications. Eventually, this pool was narrowed down to eight applications, and finally, in 1996, four were funded, with UWEB being one of them. UWEB communicated a vision of a partnership between engineering, medicine, biology, and the medical device industry with the mission to advance the performance of biomaterials used in implanted medical devices. At our high point, UWEB had 60 companies in partnership to advance biomaterials. The strategic plan for UWEB, as required by the NSF, should have three planes: Basic Science, Enabling Technology, and Engineered Systems. The strategic plan for UWEB in its last 5 years is presented in Figure 2. The basic science plane focused on the biology of molecules involved in healing. The technology plane featured technologies to bring plane one strategy to the surface of biomaterials. Plane three imagined bringing plane two technologies to real-word implants and medical devices. A key focus throughout the UWEB program was reducing or eliminating implanted device fibrosis. If the FBR could be mitigated, implanted sensors could function long-term in the body, drug delivery devices would not be compromised in their release rates by a dense capsule, glaucoma drains might not fail due to fibrosis, and breast implants would not be impacted by dense capsules—overall, most implant devices could function in an improved fashion without the FBR.

From 1996 to 2007, important discoveries and technologies originated from the UWEB ERC. Also, a few companies were spun off, and some are still in operation. Just a few of the developments nucleated by UWEB will be highlighted in this article. The emphasis is on linking the fundamental scientific discoveries to the technologies they spawned and the impact derived.

## 2. UWEB Discoveries and Technologies in Healing and Regeneration

It was critical to learn exactly which parameters directed implanted materials down a pro-healing, non-fibrotic pathway. Clearly, porosity was important. However, Brauker’s published study on this work used commercially available materials with irregularly shaped pores and a broad distribution of pore sizes [5]—it seemed impossible to tell which pore size was most important. With inspiration from the lost wax method, a fabrication method used since the Roman Empire, we developed a method to make polymeric materials with interconnected pores all of the same pore size. The template for the pores was uniform-sized beads [6,7].

**Figure 3:**
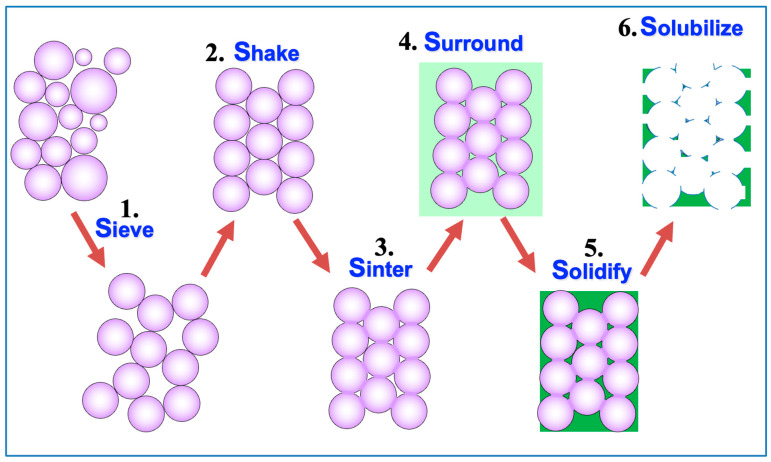
The “6S” process—Sieve/Shake/Sinter/Surround/Solidify/Solubilize—to make porous materials with interconnected pores where all pores are the same size.

In 1999, UW Chemical Engineering Ph.D. student Andrew Marshall was assigned the task of developing porous materials where all pores were the same size and then exploring the biological properties of these unique porous materials. The first uniform-sized beads we tried as pore templates were glass, but chemistries to remove the beads required unacceptably harsh conditions. We learned of poly(methyl methacrylate) (PMMA) beads that were used in fingernail salons to fabricate false nails. These beads of uncrosslinked PMMA were available in large quantities at low prices but were comprised of a broad range of bead sizes [7]. We purchased a sieving apparatus to fractionate beads into relatively tight size ranges. With those fractionated beads, we developed the “6S” process to make these porous materials (Figure 3) and, indeed, 6S led to success.

We used the 6S process to synthesize unidimensional-pore hydrogels in a range of pore sizes. These were implanted subcutaneously in mice. Upon explantation and histology, a surprising observation was made. If pores were in the 30–40 micron range, the biological reaction was characterized by little fibrosis, excellent vascularity, and high degrees of cellularity. If the pores were larger or smaller than 30–40 microns, considerable fibrosis, low cellularity, and low vascularity were noted [8,9]. In collaboration with our University of Washington Division of Dermatology, 6S rods were implanted percutaneously in the backs of mice. After one month, both the dermis and epidermis regenerated through the porous structure [10]. In collaboration with the Department of Ophthalmology, 6S implants in the sclera of rabbits were performed [11]. The extensive migration of healthy scleral cells into the porous structure was noted. Implantation in heart muscle showed that the porous structure healed with heart stroma rather than fibrotic tissue [8]. Other implant sites similarly showed reconstructive healing in contrast to avascular, fibrotic healing. The healing was independent of the biomaterial used to fabricate the 6S structure—silicone rubber, polyurethane, hydrogel, and fibrin were all fabricated with the 6S architecture and all healed similarly. Incidentally, one of the core UWEB hypotheses was that we could develop biomaterials with surface chemistries that would direct healing. With 6S, the surface chemistry did not matter—only the pore size was important.

The excellent healing noted with the 6S material led to the formation of the company Healionics, Inc. (Wavre, Belgium) (2007) and the licensing from the University of Washington of the 6S structure [12]. Healionics branded the material as STAR™ (Sphere-Templated Angiogenic Regenerative). In 2010, Healionics spun off a company that opened its doors in Belgium, iSTAR Medical. iSTAR developed the 6S material into a minimally invasive glaucoma drain device. The ability of the 6S structure to heal into the sclera with vascularity and without fibrosis has led to European Union (EU) approval for the device and an ongoing clinical trial in the US. Healionics has continued to advance the 6S concept, bringing it to vascular access for hemodialysis patients. Arterio-venous (AV) vascular grafts now used for dialysis patients, primarily made from expanded polytetrafluoroethylene (ePTFE), fail at roughly 50% in one year due to hyperplasia and/or thrombosis. Healionics’ scientists hypothesized that the contractive foreign body capsule (FBC) was compressing the ePTFE graft, reducing the inner diameter and thus increasing its propensity to thrombose. They coated the external surface of the graft with the STAR material in a granular form. Upon implantation in sheep, ultrasound imaging supported the contractive hypothesis, showing that coated grafts had larger luminal diameters and a more pulsatile appearance. Healionics has taken the graft coated with the STAR material (branded STARGRAFT™) to clinical trials. The most recent results show unprecedented patency at 1 year in comparison to ePTFE grafts without the STAR material outer surface. A 2020 start-up from our UW group, Kuleana Technology, Inc. (Seattle, WA, USA), is now advancing the 6S vascular graft concept with an all-polyurethane graft that is constructed with a continuous 6S interconnected pore structure from the outer wall through to the lumen. Consistent with the hypothesis that the endothelial lining of blood vessels is seeded by transmural capillaries in-growing from the external tissue space to the lumen [13], initial implantations in sheep support this idea. We have found substantially higher endothelial coverage in our 6S-based polyurethane graft than in conventional porous Teflon grafts [14]).

Through the work on 6S porous materials dominated UWEB studies of implant healing, UWEB explored other routes to improve healing responses through the control of biomaterial shapes and morphology. For example, using electrospinning fibers of different diameters, Prof. Joan Sanders and her group have demonstrated that fibers less than 2 µm in diameter did not trigger an FBR [15]. Importantly, for our conceptualization of the biological reaction to materials at that time, the surface charge on the fibers made no difference in the FBR healing—only the fiber geometry (diameter) was important in healing.

Another route to biomaterials exhibiting non-fibrotic healing has its roots in protein adsorption studies launched in our early NIH cell interactions grant with follow-up in NESAC/BIO and the UWEB program. In UWEB, we continued to ask, “why do most biomaterials (hydrophilic, hydrophobic, metallic, ceramic, glass, polymeric, hydrogel, etc.) lead to an almost identical FBR?” We hypothesized that the denatured proteins that adsorbed on the biomaterials were recognized as foreign by the body—this denatured, mixed protein film was not a part of normal biology, and thus, it was attacked, leading to the FBR. This has led to the hypothesis that if all protein could be inhibited from adsorbing, the body might not “see” the implant, and thus, it would not be attacked.

Our first “protein-resistant” or non-fouling materials were based on poly(hydroxyethyl methacrylate) [p(HEMA)]. These showed reduced protein adsorption and, typically, a thinner FBC but not an elimination of the FBR. In the early 1980s, poly(ethylene glycol) (PEG) was becoming well-recognized as a material for inhibiting protein adsorption [16]. We developed glow–discharge plasma methods to deposit PEG on surfaces [17]. Though they showed much-reduced protein adsorption, they still fibrosed upon implantation. Around 2000, Professor Shaoyi Jiang arrived at our University of Washington Chemical Engineering Department and introduced us to zwitterionic hydrogels. His development of acrylate polymers and acrylamide polymers with zwitterionic carboxybetaine side-chains demonstrated that it is possible to fabricate solid surfaces that adsorb near zero levels of protein, even after longer immersions in undiluted blood plasma [18]. Furthermore, those surfaces can be implanted in animals for three months [19] or even one year [20] with no FBR, supporting the hypothesis that adsorbed proteins trigger the FBR. In a spin-off company from a recent project (2017–present) to create portable and wearable hemodialysis systems, Kuleana Technology, Inc. (Seattle, WA, USA), we make extensive use of these carboxybetaine polymers invented during the UWEB years [21].

The UWEB program funded still another pathway to control the response of the body to implanted biomaterials and medical devices. We hypothesized that specific proteins oriented correctly (in contrast to a mix of denatured proteins) could direct the biological reaction down a reconstructive pathway, leading to a vascularized, integrative healing without the fibrotic scar. Which protein or proteins might do this, and how to deliver those protein signals? These were key questions that we asked when launching this area of study in UWEB.

Early in the UWEB program, we focused on fibrinogen, a protein with numerous functions and biological interactions. Though adsorbed fibrinogen’s importance for blood interactions was clearly demonstrated [22], we found no effect of adsorbed fibrinogen on the FBR.

We were fortunate to have on our team biochemists with expertise in a class of proteins that seemed promising candidates for modulating the healing reaction—matricellular proteins. Matricellular proteins were defined at the University of Washington in the 1980s [23]. The matricellular protein concept originated from UW professor Paul Bornstein, a UWEB investigator. When Dr. Bornstein started his work in the Biochemistry Department at the UW around 1973, much of the department was focused on blood coagulation proteins. Proteins such as collagen and other extracellular matrix (ECM) proteins were thought of as inert “filler” between cells and not as proteins worthy of serious study. Bornstein was a pioneer in defining the biologically active role of collagens and, with his colleague, Prof. Helene Sage, elucidating the key interactions between matricellular proteins and collagens. Matricellular proteins that were explored in these seminal studies included thrombospondins, osteopontin, tenacin, fibronectin, vitronectin, and SPARC (secreted protein acidic and rich in cysteine). The importance of collagen type I for delivering biological signals was highlighted in a paper that demonstrated that collagen type I could bind to more than 50 different biomolecules [24]. It was noted that collagen binds matricellular proteins, and the matricellular protein–collagen complex has unique biological activity and biological signaling. Examples of three important studies focusing on matricellular proteins that were funded by the UWEB ERC follow.

**Figure 4:**
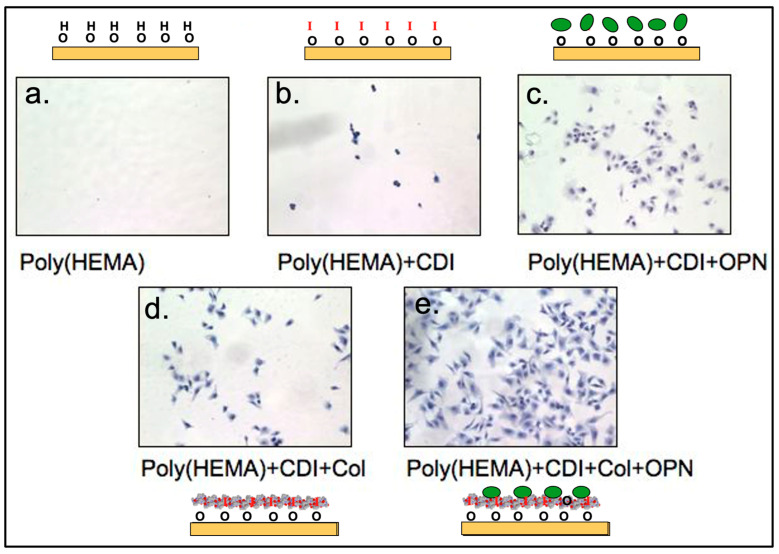
A schematic representation of the conclusions on bovine aortic endothelial cell (BAEC) adhesion to surface from Martin, et. al, 2004 [25]. Letter “I” in red represents carbonyl diimidazole (CDI). Green ovals represent osteopontin molecules. (**a**) poly(hydroxyethyl methacrylate) (PHEMA) surface do not support BAEC attachment. (**b**) PHEMA surfaces treated with CDI support little BAEC adhesion. (**c**) Osteopontin immobilized to PHEMA -OH groups exhibits intermediate levels of BAEC attachment. (**d**) Collagen type I immobilized with CDI to PHEMA exhibits intermediate levels of BAEC attachment. (**e**) The collagen immobilized surfaces from panel (**d**), if exposed to osteopontin solution, then exhibits vigorous BAEC attachment.

**Osteopontin:** A study that highlighted the enhanced biological activity of a matricellular protein when coupled with collagen looked at the interaction between collagen type I and osteopontin [25]. We surmised that collagen type I might be especially effective for delivering protein signals because nature made extensive use of collagen–biomolecule interactions. Also, from a bioengineering standpoint, collagen has many positive attributes: it was axially symmetric (no up or down side-, a rigid-rod), durable and robust, inexpensive, accepted by regulatory authorities, and binds many different molecules. The study described schematically in Figure 4, looked at bovine aortic endothelial cell (BAEC) adhesion to a series of surfaces. BAEC demonstrated little or no adhesion to poly(hydroxyethyl methacrylate) (PHEMA) surfaces. If osteopontin was immobilized non-specifically using carbonyl diimidazole (CDI) to the PHEMA through the coupling of one of the many surface amine groups on the osteopontin molecule with the hydroxy groups on the PHEMA, a low level of BAEC adhesion was noted [25]. If type I collagen was immobilized using the CDI to PHEMA, a low level of adhesion of the BAEC was also observed. However, if type I collagen was immobilized using the CDI to the PHEMA surface and then exposed to an osteopontin solution, vigorous BAEC adhesion was noted. We hypothesized that the type I collagen bound the osteopontin in a biospecific configuration that oriented the RGD (arginylglycylaspartic acid) cell–adhesion binding motif of osteopontin so as to be accessible to the integrin receptor on the cell surface (note: all surfaces with osteopontin had similar surface levels of the protein). Thus, type I collagen surfaces can be effective in delivering biological signals [25].

**Thrombospondin 2**: A matricellular protein study funded through the UWEB program considered thrombospondin 2 (TSP2) [26]. TSP2 is not a structural protein but rather works in conjunction with the ECM and modulates biological reactions. Mice engineered to not produce TSP2 (knockout mice) show evidence of abnormal collagen fibrillogenesis and increased dermal and subdermal vascular density. Because these processes are prominent in the FBR, we hypothesized that the implant site collagenous capsule and the degree of vascularization might be altered in these knockout mice. Silicone rubber (PDMS) and hydrophilic surface-modified PDMS were implanted subcutaneously in normal mice and TSP-2 knockouts (Table 1). The study strongly implicated TSP2 as the agent directing the FBR toward the avascular, dense, fibrotic capsule. In a follow-up experiment, anti-sense RNA was used in normal mice to inhibit the local production of TSP2. By minimizing local TSP2, a more vascularized foreign body capsule was noted. These experiments suggest that minimizing TSP2 locally at implant sites could be an approach to improve implant healing and integration. Interestingly, and consistent with other observations made in the UWEB program, silicone rubber and hydrophilic-modified silicone rubber healed almost indistinguishably with similar foreign body capsules in all in vivo experiments.

**SPARC**: Another matricellular protein studied in the UWEB program was SPARC, also called osteonectin. SPARC is a moderator of cell–ECM interactions. SPARC-null mice showed accelerated wound closure and an altered deposition of collagen [27]. A UWEB team, headed by Dr. Helen Sage, asked whether SPARC might influence the FBR to biomaterial implants [28]. PDMS disks and cellulose filters were implanted into wild-type and SPARC knockout mice. In wild-type animals, high levels of SPARC were found in the ECM and cells of the foreign body capsules surrounding the implants. SPARC knockout mice were found to have a thinner foreign body capsule compared to that observed in wild-type mice. A significant reduction in capsular vascular density was observed with the PDMS implants in the SPARC knockout animals. Collagen fibers in the SPARC knockout mice foreign body capsules were smaller and more uniform in size than those observed in the wild-type mice and less mature than in wild-type animals, as observed after picrosirius-red staining. Overall, there was decreased capsular thickness, suggesting that control of SPARC was still another route to moderate the FBR [28].

**Figure 5:**
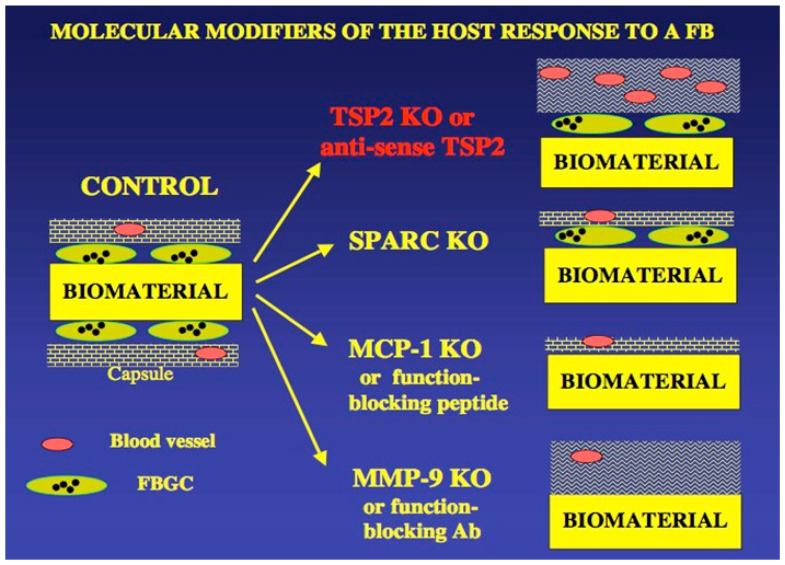
In a diagram assembled by Dr. Themis Kyriakidas, a postdoctoral fellow in the UWEB ERC ~2002–2005 (now Professor at Yale University), the ability of matricellular proteins to modulate the FBR is schematically illustrated. The diagram on the left side of Figure 5 illustrates the classic FBR with its dense, collagenous capsule containing few blood vessels (the foreign body capsule) and giant cells (fused macrophages) at the interface of the biomaterial. Matricellular proteins are associated with the thickness of the capsule, its morphology, the collagen density, the blood vessel density in the capsule and interfacial giant cells.

A useful diagram that summarizes UWEB studies on matricellular proteins and the FBR is shown in Figure 5. This summary diagram highlights an important conclusion from UWEB studies on the healing of implanted biomaterials and medical devices—the non-specific signals sent by interactions of synthetic biomaterials with the body have little impact on the FBR, but biospecific signals (proteins, other biological molecules) can indeed alter and ameliorate the course of the FBR.

### Additional UWEB Technologies Impacting Biomaterials

Material and biology research in UWEB was primarily focused on impacting how biomaterials influenced healing upon implantation. However, an additional, substantial body of work led to new technologies to support the central UWEB mission. We studied and advanced methods for the immobilization of factors, the delivery of blocking agents (antibodies, anti-sense DNA, siRNA, etc.), and novel controlled-release systems. Surface modification was important throughout the program (a continuation of work started in NESAC/BIO). Surfaces designed to possess near-zero non-specific protein adsorption (*stealth biomaterials*) and surfaces to present immobilized biomolecules were developed. Controlled release systems were also important. The issues of infection and biofilms led to studies of strategies to address these complications that plagued patients receiving implants and, by association, the biomedical device industry making the implants and devices.

## 3. Additional UWEB Contributions to Education/Outreach and Society

In addition to impactful technical contributions and consistent with the NSF program requirements for ERCs, UWEB was assessed by the NSF based on contributions to education/outreach. Numerous programs were launched by UWEB that engaged thousands of students, exposing them to the exciting fields of biomaterials and medical devices.

**SET-UP (Scholarship in Engineering Training in the UWEB Program)**: The SET-UP program contributed to our efforts to bring students from historically underrepresented groups into science and engineering. SET-UP brought six Seattle African American Academy (AAA) middle-school students onto the UW campus each quarter for an eight-week series, during which they learned from UWEB scientists (graduate students, postdocs, and professors) and explored topics related to bioengineering and biomaterials (examples: making hydrogel contact lenses, growing crystals, learning about DNA base pairing by a “DNA dance”). Over 120 “young scholars” were impacted by SET-UP. A yearly “graduation day” in June recognized the AAA SET-UP scholars and brought their parents to the AAA to see the students honored.

**UWEB visits to Washington State schools:** From August 2006 to June 2007, through school visits, UWEB volunteers made over 80 presentations, directly reaching over 2500 students in 42 different K-12 schools across Washington State. Most of the schools visited represent urban or rural school districts consisting of students in populations underrepresented in science and engineering. Through on-campus visits and campus-wide events, thousands more have been exposed to the science of biomaterials.

**Guy Simplant—The Case of the Missing Hand:** In collaboration with our UW School of Art, we developed a CD-ROM-based computer game where a “James Bond-like” secret agent/detective, Guy Simplant, solved mysteries involving biomaterials (how to replace a lost hand). Thousands of copies of the game were distributed as CD-ROMs and later in a downloadable form from the UWEB website.

**Lab Experience for High School Students (LEHSS):** LEHSS was developed to target high-school juniors with an interest in science and engineering. The primary goal of this program was to encourage a diverse student population to pursue a career in science/engineering and to acquaint them with exciting research in engineered biomaterials at the University of Washington. UWEB provided the opportunity for selected students from ten local high schools to gain experience in laboratory research. The students were on campus between 1:00 p.m. and 5:00 p.m. each day for two weeks, during which time they attended brief lectures and worked in the laboratory in pairs on actual research projects. The molecular biology component was aimed at understanding tissue culture, DNA transfection into bacteria, and control over ligands and receptors impacting implanted biomaterials.

**Science for Success (SFS):** The SFS was a summer science outreach program for economically disadvantaged and underrepresented minority high-school students. The UWEB-led program was funded by the Medtronic Foundation, Inc.(Minneapolis, MN, USA), the NSF, and the National Nanotechnology Infrastructure Network (NNIN). The primary objective of the SFS program was to encourage promising underrepresented and economically disadvantaged high-school students towards a career in science/engineering as well as to acquaint them with exciting research in biomaterials, bioengineering, molecular biotechnology, mathematics, and environmental health. A total of 84 students participated in six summer SFS programs (2001–2006). Sessions included lectures, hands-on laboratory experiences, and field trips focused on topics such as engineered biomaterials, environmental and human biology, molecular biotechnology, and quantitative methods using computers.

**Science Teachers Institute (STI):** Middle- and high-school teachers throughout the State of Washington were able to experience bioengineering developments firsthand at the UWEB Science Teachers Institute (STI) and shared their learning with students through curriculum development and the STI materials loan program. Starting with its inception in the summer of 1999, the STI grew to a comprehensive training and curriculum program serving teachers statewide. Each of the three summer institutes was held for three days. Teachers received content knowledge, lab experience, and the materials management skills necessary to make their instructional units powerful learning experiences for students. Kits were developed so teachers could loan them and have access to materials not readily available in public schools. The success of the UWEB kit loan program was demonstrated by the steady demand and use over seven years. The kits were checked out 48 times and used with more than 4500 students. In conjunction with the kit loan program, UWEB faculty, staff, and students gave presentations to more than 80 classrooms, reaching over 2500 students from kindergarten through community college. Educational units through the STI included all the curriculum and equipment needed to complete a month of instruction focused on angiogenesis, cochlear implantation, and tissue engineering. More specifically, the units were “Build Me Up, Scotty!” (tissue engineering, stem cell research and ethics); “Stick This in Your Ear!” (the science and ethics behind cochlear implants); “What’s Growing On?” (Integrated mathematics and science through the study of fractals and angiogenesis as they relate to the growth of organisms in the living world).

**Youth Take Heart (YTH):** In 2003, a USD $1.55 million Science Education Partnership Award (SEPA) from NIH to UWEB Education and Outreach permitted the development of YTH in partnership with the non-profit Hope Heart Institute and the Washington MESA (Math Engineering, Science Achievement) program. The goal of the YTH program was to develop an educational plan examining cardiovascular health aimed at students in middle school (grades 5–8). YTH was intended to introduce students, especially students from historically underrepresented groups, to careers in health and science and to educate them about good heart health practices. Middle-school teachers received materials (kits) and training so that they could educate their students about heart anatomy, physiology, nutrition, lifestyle choices that impact heart health and bioengineering. The curriculum took six weeks to complete. This program not only contained science content but also taught students how to think like scientists as they consider how to best design an artificial blood vessel. Also, another Guy Simplant digital adventure was created, “the Case of the Ailing Heart”.

**Research Experience for Community College Students (RECCS):** In Year 6 of the UWEB program, we launched RECCS. This program was aimed at providing enrichment and exposure for community college students by bringing them into university research. We enrolled six local community college students in a 10-week summer program in an alliance with Seattle Community College. RECCS included workshops, journal clubs, and other seminars to supplement the research experience.

## 4. Summary of UWEB Accomplishments

Prior to UWEB, there was little appreciation of the negative aspects of the FBR. A biomaterial would be considered “biocompatible” if it was walled off from the body by a thin FBR capsule with low cellularity. Conversely, a premise of early biomaterials research, never quite proven, was that the chemistry of the biomaterial impacts the way it heals. A 1977 study, prescient in its appreciation of the problem that all biomaterials without toxic leachables healed with the same FBR, was by Robert Bagnall [29]. But following that work, little was written on the triggers of the FBR or methods to ameliorate the FBR. James Anderson’s 1984 publication clarifying the role of the macrophage in the FBR was an important milestone in the field [30]. UWEB and its focus on eliminating or minimizing the FBR probably stimulated the field, and now there are many researchers looking to “tame” the FBR. A biomaterials sub-field has arisen, immunoengineering, and there are now many published studies focused on the control of the healing around implanted biomaterials [31,32,33,34,35,36,37,38,39,40].

UWEB funding and UWEB investigators, over 11 years, produced 511 articles in peer-reviewed journals, 379 publications in conference proceedings, 54 inventions were disclosed, and 35 patent applications were filed. UWEB invented the term “engineered biomaterial” to distinguish engineered biomaterials from “off-the-shelf” commodity materials that dominated biomaterials in the decades prior to UWEB. A number of UWEB-funded studies led to the commercialization of developments. UWEB students and postdocs have gone on to professorships, leadership roles in medical device companies, positions in the FDA, and other jobs educating the public about biomaterials. Overall, the NSF investment in UWEB paid back impressive dividends. And, from the standpoint of the PI, it was 11 grueling years, but so very worth the effort!

## Figures and Tables

**Table 1:** Vascular density and capsule thickness of foreign body capsules in control and TSP2-null mice (4-week implantation).

			Capsule Thickness facing:	
**Genotype**	**Material**	**Vessels/Capsule**	**Dermis (µm)**	**Body (µm)**
(Thbs2): +/+	PDMS	10 ± 10	55 ± 8	24 ± 3
(Thbs2): +/+	Ox-PDMS	12 ± 8	58 ± 7	25 ± 4
(Thbs2): −/−	PDMS	100 ± 20	91 ± 7	36 ± 5
(Thbs2): −/−	Ox-PDMS	90 ± 14	95 ± 8	38 ± 5

Data from reference [26]. Thrombospondin 2 gene (Thbs2): +/+ = gene active; −/− + gene knocked out. Poly(dimethyl siloxane)(PDMS) and oxidized PDMS (Ox-PDMS).

## References

[1] Ratner B.D. (1993). New ideas in biomaterials science—A path to engineered biomaterials. J. Biomed. Mater. Res..

[2] Ross R. (1968). The fibroblast and wound repair. Biol. Rev..

[3] Ross R., Balazs E.A. (1970). Wound healing—A review. Chemistry and Molecular Biology of the Intercellular Matrix.

[4] Benditt E.P., Benditt J.M. (1973). Evidence for a monoclonal origin of human atherosclerotic plaques. Proc. Natl. Acad. Sci. USA.

[5] Brauker J.H., Carr-Brendel V.E., Martinson L.A., Crudele J., Johnston W.D., Johnson R.C. (1995). Neovascularization of synthetic membranes directed by membrane microarchitecture. J. Biomed. Mater. Res..

[6] Marshall A.J., Irvin C.A., Barker T., Sage E.H., Hauch K.D., Ratner B.D. (2004). Biomaterials with tightly controlled pore size that promote vascular in-growth. Polym. Prepr..

[7] Marshall A., Ratner B.D. (2005). Quantitative characterization of sphere-templated porous biomaterials. AIChE J..

[8] Madden L.R., Mortisen D.J., Sussman E.M., Dupras S.K., Fugate J.A., Cuy J.L., Hauch K.D., Laflamme M.A., Murry C.E., Ratner B.D. (2010). Proangiogenic scaffolds as functional templates for cardiac tissue engineering. Proc. Natl. Acad. Sci. USA.

[9] Sussman E.M., Halpin M.C., Muster J., Moon R.T., Ratner B.D. (2014). Porous Implants Modulate Healing and Induce Shifts in Local Macrophage Polarization in the Foreign Body Reaction. Ann. Biomed. Eng..

[10] Fukano Y., Usui M.L., Underwood R.A., Isenhath S., Marshall A.J., Hauch K.D., Ratner B.D., Olerud J.E., Fleckman P. (2010). Epidermal and dermal integration into sphere-templated porous poly(2-hydroxyethyl methacrylate) implants in mice. J. Biomed. Mater. Res. Part A.

[11] Teng W.Q., Long T.J., Shen T., Ratner B. (2014). A tough, precision-porous hydrogel scaffold: Ophthalmologic applications. Biomaterials.

[12] Ratner B.D., Marshall A. (2008). Novel Porous Biomaterials. U.S. Patent.

[13] Clowes A.W., Kirkman T.R., Reidy M.A. (1986). Mechanisms of arterial graft healing—Rapid transmural capillary ingrowth provides a source of intimal endothelium and smooth muscle in porous PTFE prostheses. Am. J. Pathol..

[14] Zhen L., Quiroga E., Creason S.A., Chen N., Sapre T.R., Snyder J.M., Lindhartsen S.L., Fountaine B.S., Barbour M.C., Faisal S. (2024). Immunomodulatory Porous Regenerative Scaffolds for in situ Vascular Engineering. bioRxiv.

[15] Sanders J.E., Cassisi D.V., Neumann T., Golledge S.L., Zachariah S.G., Ratner B.D., Bale S.D. (2003). Relative influence of polymer fiber diameter and surface charge on fibrous capsule thickness and vessel density for single-fiber implants. J. Biomed. Mater. Res..

[16] Merrill E.W., Salzman E.W. (1983). Polyethylene oxide as a biomaterial. ASAIO J..

[17] Lopez G.P., Ratner B.D., Tidwell C.D., Haycox C.L., Rapoza R.J., Horbett T.A. (1992). Glow discharge plasma deposition of tetraethylene glycol dimethyl ether for fouling-resistant biomaterial surfaces. J. Biomed. Mater. Res..

[18] Zhang Z., Zhang M., Chen S., Horbett T., Ratner B., Jiang S. (2008). Blood compatibility of surfaces with superlow protein adsorption. Biomaterials.

[19] Zhang L., Cao Z., Bai T., Carr L., Ella-Menye J.-R., Irvin C., Ratner B.D., Jiang S. (2013). Zwitterionic hydrogels implanted in mice resist the foreign-body reaction. Nat. Biotechnol..

[20] Dong D., Tsao C., Hung H.-C., Yao F., Tang C., Niu L., Ma J., MacArthur J., Sinclair A., Wu K. (2021). High-strength and fibrous capsule–resistant zwitterionic elastomers. Sci. Adv..

[21] Himmelfarb J., Ratner B.D. (2024). An Innovation Ecosystem. J. Am. Soc. Nephrol..

[22] Wu Y., Simonovsky F.I., Ratner B.D., Horbett T.A. (2005). The role of adsorbed fibrinogen in platelet adhesion to polyurethane surfaces: A comparison of surface hydrophobicity, protein adsorption, monoclonal antibody binding, and platelet adhesion. J. Biomed. Mater. Res. A.

[23] Murphy-Ullrich J.E., Sage E.H. (2014). Revisiting the matricellular concept. Matrix Biol..

[24] de Lullo G.A., Sweeney S.M., Korkko J., Ala-Kokko L., San Antonio J.D. (2002). Mapping the ligand-binding sites and disease-associated mutations on the most abundant protein in the human, type I collagen. J. Biol. Chem..

[25] Martin S.M., Schwartz J., Giachelli C.M., Ratner B.D. (2004). Enhancing the biological activity of immobilized osteopontin using a type-1 collagen affinity coating. J. Biomed. Mater. Res..

[26] Kyriakides T.R., Leach K.J., Hoffman A.S., Ratner B.D., Bornstein P. (1999). Mice that lack the angiogenesis inhibitor, thrombospondin 2, mount an altered foreign body reaction characterized by increased vascularity. Proc. Natl. Acad. Sci. USA.

[27] Bradshaw A.D., Sage E.H. (2001). SPARC, a matricellular protein that functions in cellular differentiation and tissue response to injury. J. Clin. Investig..

[28] Puolakkainen P., Bardshaw A.D., Kyriakides T.R., Reed M., Brekken R., Wight T.N., Bornstein P., Ratner B., Sage H. (2003). Compromised production of extracellular matrix in mice lacking secreted protein, acidic and rich in cysteine (SPARC) leads to a reduced foreign body reaction to implanted biomaterials. Am. J. Pathol..

[29] Bagnall R.D. (1977). An approach to the soft tissue/synthetic material interface. J. Biomed. Mater. Res..

[30] Anderson J.M., Miller K.M. (1984). Biomaterial biocompatibility and the macrophage. Biomaterials.

[31] Crawford L., Wyatt M., Bryers J., Ratner B. (2021). Biocompatibility Evolves: Phenomenology to Toxicology to Regeneration. Adv. Heal. Mater..

[32] Chu C., Liu L., Rung S., Wang Y., Ma Y., Hu C., Zhao X., Man Y., Qu Y. (2020). Modulation of foreign body reaction and macrophage phenotypes concerning microenvironment. J. Biomed. Mater. Res. Part A.

[33] Major M.R., Wong V.W., Nelson E.R., Longaker M.T., Gurtner G.C. (2015). The foreign body response: At the interface of surgery and bioengineering. Plast Reconstr. Surg..

[34] Li J., Jiang X., Li H., Gelinsky M., Gu Z. (2021). Tailoring Materials for Modulation of Macrophage Fate. Adv. Mater..

[35] Horejs C. (2021). Preventing fibrotic encapsulation. Nat. Rev. Mater..

[36] Zhen L., Creason S.A., Simonovsky F.I., Snyder J.M., Lindhartsen S.L., Mecwan M.M., Johnson B.W., Himmelfarb J., Ratner B.D. (2021). Precision-porous polyurethane elastomers engineered for application in pro-healing vascular grafts: Synthesis, fabrication and detailed biocompatibility assessment. Biomaterials.

[37] Vishwakarma A., Bhise N.S., Evangelista M.B., Rouwkema J., Dokmeci M.R., Ghaemmaghami A.M., Vrana N.E.A. (2016). Khademhosseini, Engineering Immunomodulatory Biomaterials To Tune the Inflammatory Response. Trends Biotechnol..

[38] Zhang D., Chen Q., Shi C., Chen M., Ma K., Wan J., Liu R. (2020). Dealing with the Foreign-Body Response to Implanted Biomaterials: Strategies and Applications of New Materials. Adv. Funct. Mater..

[39] Liu L., Chen G., Chao T., Ratner B.D., Sage E.H., Jiang S. (2008). Reduced foreign body reaction to implanted biomaterials by surface treatment with oriented osteopontin. J. Biomater. Sci. Polym. Ed..

[40] Bryers J.D., Giachelli C.M., Ratner B.D. (2012). Engineering biomaterials to integrate and heal: The biocompatibility paradigm shifts. Biotechnol. Bioeng..

